# Development and validation of prognostic index based on autophagy-related genes in patient with head and neck squamous cell carcinoma

**DOI:** 10.1038/s41420-020-00294-y

**Published:** 2020-07-14

**Authors:** Hao Feng, Linna Zhong, Xiangjun Yang, Qianbing Wan, Xibo Pei, Jian Wang

**Affiliations:** 1 State Key Laboratory of Oral Diseases, National Clinical Research Center for Oral Diseases, Chengdu, 610041 China; 2 Department of Prosthodontics, West China Hospital of Stomatology, Sichuan University, Chengdu, 610041 China

**Keywords:** Oral cancer detection, Genetic variation

## Abstract

Head and neck squamous cell carcinoma (HNSCC) is one of the most common cancers worldwide, accounting for almost 50% of all malignancies in developing nations. Autophagy plays a vital role in cancer initiation, malignant progression, and resistance to treatment. However, autophagy-related gene sets have rarely been analyzed in HNSCC. Hence, it is necessary to assess its clinical and pathological significance in a larger cohort of patients with HNSCC. The purpose of this study was to establish a novel autophagy-related prognostic marker for HNSCC. We screened 232 autophagy-related genes (ARGs) and identified 38 differentially expressed ARGs in The Cancer Genome Atlas (TCGA) cohorts. The prognosis-related ARGs signature, established using the univariate and multivariate Cox proportional regression models, consists of 10 ARGs that could divide patients into high-risk and low-risk groups. Survival analysis indicated that patients in the high-risk group had dramatically shorter overall survival compared with their low-risk counterparts. Cox regression analysis further confirmed the independent prognostic value of the autophagy-related signature, and the area under the receiver operating characteristic curve of the combined prognostic model was 0.722. Finally, the efficacy of autophagy-related signature was also validated by an independent cohort from the Gene Expression Omnibus (GEO) database. Collectively, we successfully constructed a novel autophagy-related signature for the prediction of prognosis in patients with HNSCC.

## Introduction

Head and neck cancer, the sixth most common type of cancer worldwide, includes the tumors in the oral cavity, pharynx, and throat^1^. Each year in the world, more than half a million patients are diagnosed as head and neck cancer, among which 325,000 individuals die of it^2^. Head and neck squamous cell carcinoma (HNSCC) accounts for nearly 95% of head and neck malignancies, and its major causative agents are human papillomavirus (HPV) infection, cigarette smoking, and alcohol consumption. Patients with these risk factors are more likely to have worse treatment outcomes and lower survival rate^3,4^. Furthermore, the poor therapeutic outcome of HNSCC is also due to the lack of effective indicators for detecting the development of tumors at the early stage. Therapeutic progress of HNSCC is achieved by the advances in the molecular field and the development of new drugs that target at molecular abnormalities. But the existing treatment targets are prone to induce resistance^5^. New treatment markers and targets are needed to achieve better prognosis. Recently, the identification of aberrant genes has been a heated topic, in which the researches on autophagy have received increasing attention.

Autophagy is a catabolic process in which cells digest their own cellular contents, including amino acids, fatty acids, nucleotides, and damaged organelles^6^. The degradation products can be transported back and recycled for general cell metabolism. The autophagosome, a cytoplasmic double-membrane structure, can be transported into lysosome and fuse with lysosome to generate the autolysosome^7^. Autophagy also plays a dual role in cancer. In the early stages of tumor formation, it exerts anti-cancer activity to protect normal cells against malignancy^8^. In contrast, during the stage of tumor progression, autophagy is often upregulated and promotes tumor cell proliferation and invasion by absorbing nutrients and energy from degrading proteins and organelles^9^. According to the present understanding, autophagy is involved in the process of antigen presentation and the development of lymphocytes, making autophagy a possible target for improving immunotherapy in HNSCC^10^. However, the relationship between autophagy and HNSCC has not been fully revealed.

In this study, we comprehensively analyzed autophagy-related genes (ARGs) in HNSCC. We collected ARGs data and identified 10 genes related to patients’ prognosis. These 10 genes could stratify patients into two groups with significant molecular and prognostic differences, suggesting a correlation with HNSCC malignancy. Hence, we constructed autophagy-related signature which was closely related to prognosis and could serve as an independent prognostic indicator in HNSCC, and used it to identify high-risk HNSCC samples. Next, functional analysis was performed in these samples and revealed elevated autophagy and lymph node metastasis related signaling pathways which were directly related to tumor malignancy and poor prognosis.

## Results

### Differentially expressed ARGs

**Fig. 1:**
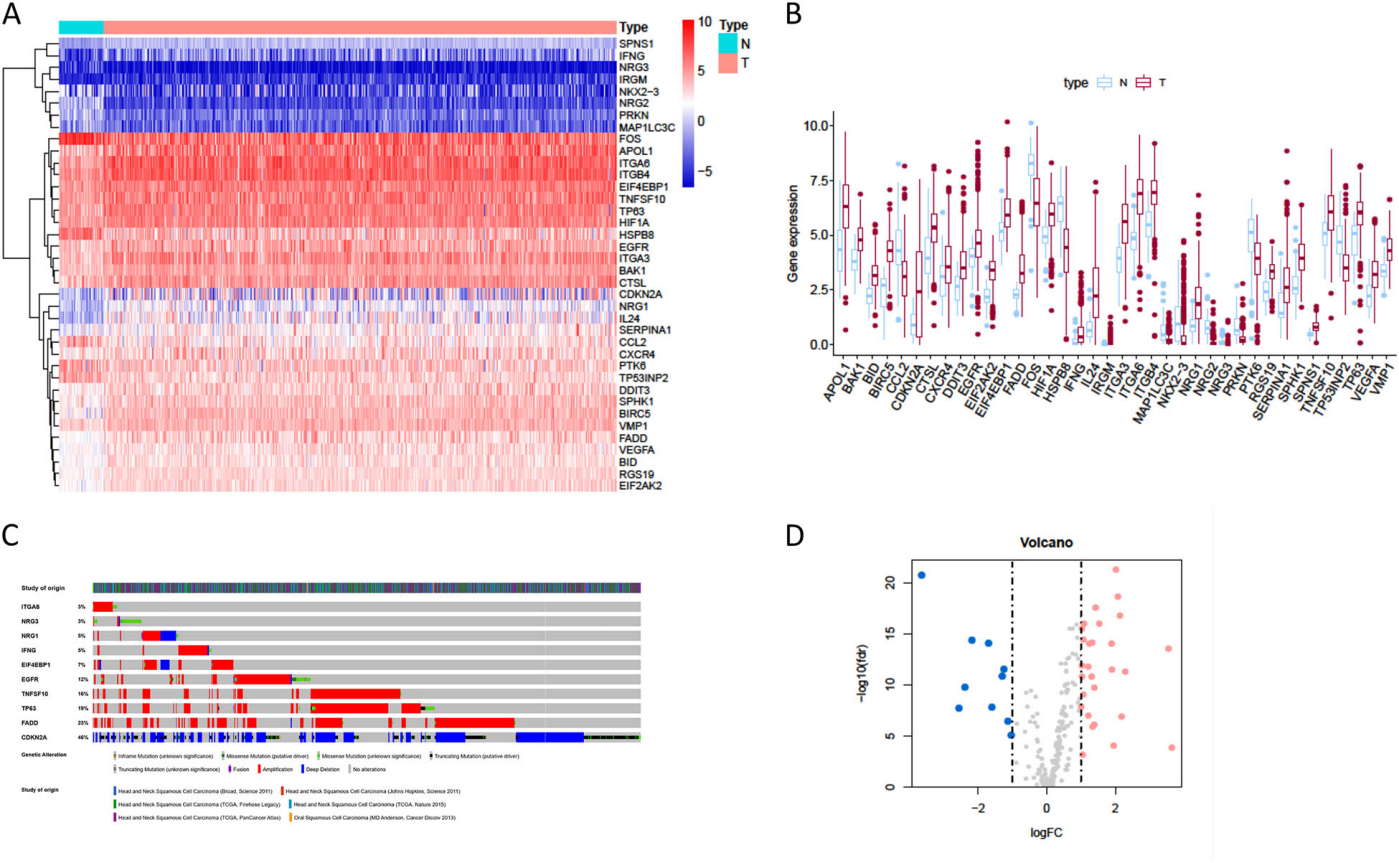
The differentially expressed ARGs. **a** The heatmaps of 38 differentially expressed ARGs. **b** The boxplot of the differentially expressed ARGs. **c** Mutations in ARGs. A total of 10 genes have a mutation rate ≥3%. **d** The volcano plot of the differentially expressed ARGs. N indicates non-tumor tissues; T indicates tumor tissues.

RNA-seq from 502 tumor tissue samples and 44 non-tumor samples were downloaded from TCGA. A total of 519 patients with primary HNSCC who were followed for more than 1 month were included in the study. The expression values of 232 autophagy-related genes were extracted from HNSCC patients. Compared with normal samples, 10 downregulated genes (NRG2, NRG3, MAP1LC3C, PRKN, HSPB8, CCL2, FOS, TP53INP2, PTK6, NKX2–3) and 28 upregulated genes (EIF4EBP1, BAK1, RGS19, HIF1A, CXCR4, CTSL, VMP1, SPNS1, TNFSF10, TP63, BID, VEGFA, SPHK1, EGFR, DDIT3, SERPINA1, EIF2AK2, ITGB4, ITGA3, APOL1, IRGM, BIRC5, FADD, ITGA6, IFNG, NRG1, IL24, and CDKN2A) were identified according to the criteria for FDR < 0.05 and [log2 (fold change)] >1 (Fig. 1a, d). Scatter plots revealed the expression patterns of these differentially expressed genes between tumor and non-tumor tissues, as shown in Fig. 1b. Due to the important clinical implications of these ARGs, we examined the genetic alterations of these genes and found that truncating mutation and missense mutation were the two most common types of mutations (Fig. 1c). A total of 10 genes had a mutation rate ≥3%, of which CDKN2A was the most frequently mutated gene (46%).

### Functional enrichment of the differentially expressed ARGs

**Fig. 2:**
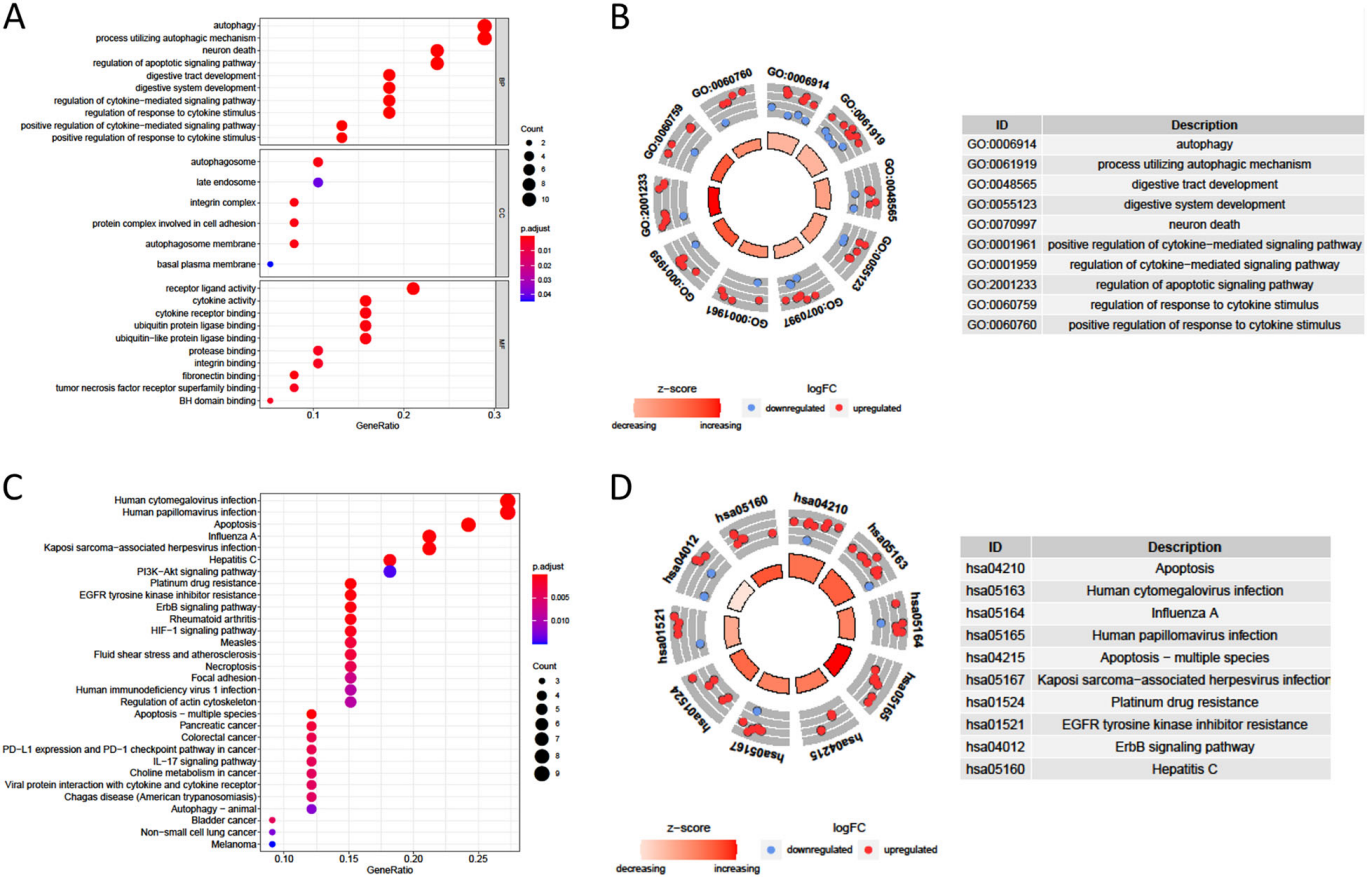
GO and KEGG analysis of differentially expressed ARGs. **a** The top 30 significant terms of GO function enrichment. BP biological process, CC cellular component, MF molecular function. **b** The GO circle shows the scatter map of the logFC of the specified gene. **c** The top 30 significant terms of KEGG analysis. **d** The KEGG circle shows the scatter map of the logFC of the specified gene. The higher the Z-score value indicated, the higher expression of the enriched pathway.

Functional enrichment analyses of the 38 differentially expressed genes offered a biological understanding of these genes. The GO term functional enrichment and the KEGG pathway enrichment analysis of these genes were summarized in Fig. 2. The top enriched GO terms in biological processes were autophagy and processes utilizing autophagic mechanisms, and those in cellular components were the autophagosome, the autophagosome membrane, and integrin complex, in terms of molecular function, genes were mostly enriched in terms of receptor ligand activity. In the KEGG pathway enrichment analysis, these genes were shown to be notably associated with pathways related to apoptosis, human cytomegalovirus infection and HPV infection. Most of the Z-scores of enriched pathways were more than zero, indicating that most of the pathways were likely to be enhanced (Figure S1B, C, supporting information).

### Construction of a signature predicting prognosis in the cohorts using the 10 autophagy-related genes

Prognostic ARGs with statistical significance in the above univariate analyses were further included in the subsequent multivariate analyses. A total of 10 genes were significantly associated with prognosis after multivariate analysis. According to the multivariate Cox proportional hazards regression model, we obtained the expression coefficient of each independent risk gene. Our prognostic model for predicting prognosis based on the 10 genes was formed using the following formula: prognosis index (PI) = (0.284 × expression level of LAMP1) + (0.355 × expression level of ATG5) + (−0.357 × expression level of NKX2–3) + (−0.269 × expression level of CFLAR) + (−0.722 × expression level of CAPN10) + (0.398 × expression level of SAR1A) + (−0.499 × expression level of MAP2K7) + (0.298 × expression level of RAB24) + (0.466 × expression level of ATIC) + (0.426 × expression level of ST13).

**Fig. 3:**
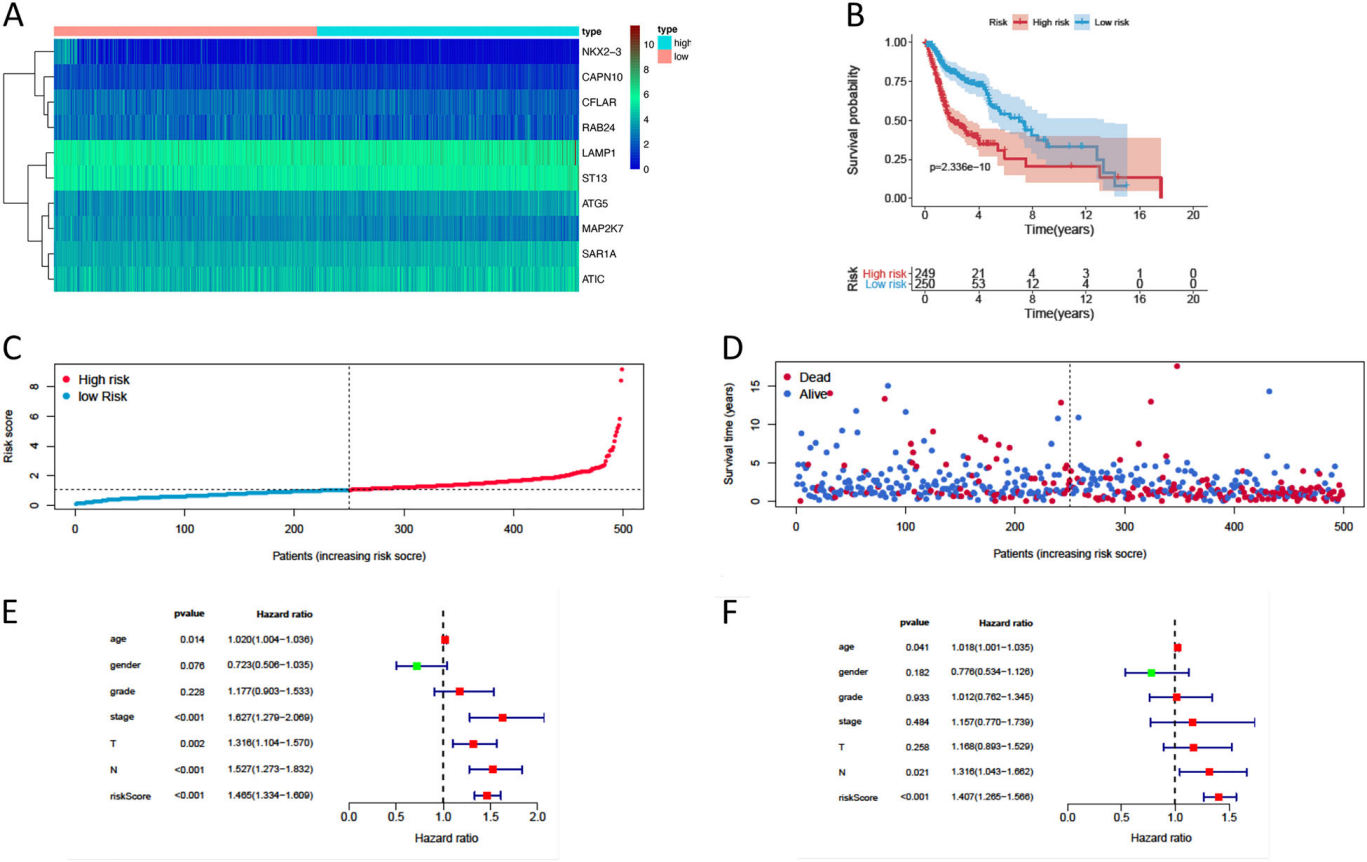
The development of a prognostic index based on ARGs. **a** Heatmap of the expression profile of the ten ARGs. **b** Kaplan–Meier curves of OS in the high-risk and the low-risk groups stratified by the autophagy-related signature in the cohorts. **c** The number of patients in different risk groups. **d** Survival status of patients in different groups. **e** A forest plot of univariate Cox regression analysis in the cohorts. **f** A forest plot of multivariate Cox regression analysis in the cohorts.

**Fig. 4:**
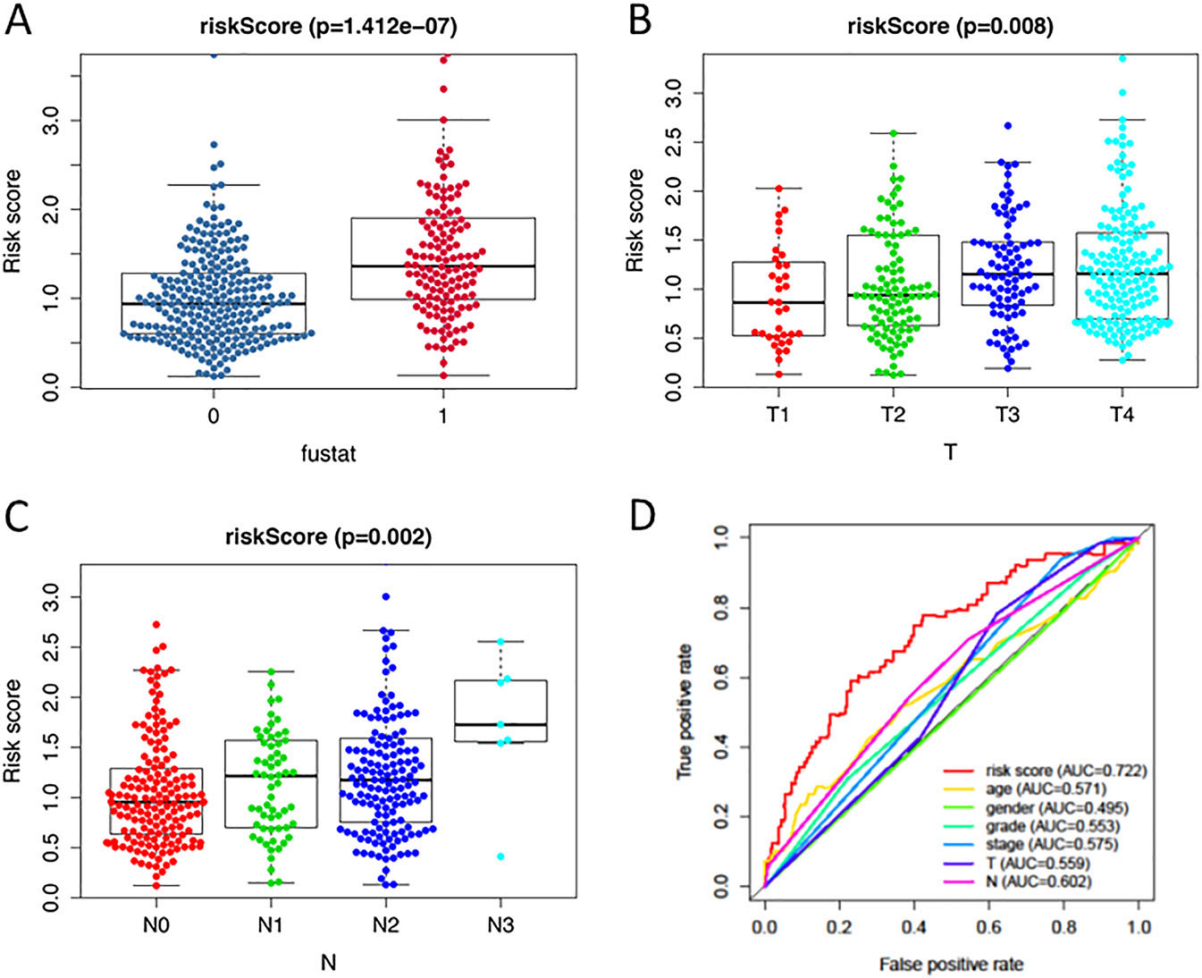
The autophagy-related signature in the cohorts. **a** The autophagy-related signature in the cohorts stratified by survival outcome. **b** The autophagy-related signature in the cohorts stratified by tumor stages. **c** The autophagy-related signature in the cohorts stratified by lymph node metastasis. **d** The ROC analysis of OS for the signature and the clinicopathologic parameters.

We then calculated the risk scores of each patient and used the median risk value as a cut-off point to classify patients into the high-risk group (*n* = 249) and the low-risk group (*n* = 250). The heatmap of these 10 signature-related genes and the Kaplan–Meier curve depending on risk scores were also displayed (Fig. 3a–d). A significant difference in the survival rate between the high-risk group and the low-risk group was observed. Patients in the high-risk group had a shorter OS than patients in the low-risk group (5-year survival rate = 30.5% vs. 58.16%, *p* < 0.001). ROC curves of OS were used to reveal the predictive performance of the ten-gene risk signature (Fig. 4d). The AUC value of the signature was 0.722, obviously higher than those associated with age, gender, grade, tumor stage, tumor T stage, and tumor N stage. These results indicated that the risk signature had a better ability to predict the survival of HNSCC patients than clinical factors.

### The autophagy-related signature is an independent prognostic factor for HNSCC patients

Clinicopathological analyses were performed to explore the associations between clinical parameters and the risk signature (Fig. 4a–c). The results showed that the signature was associated with tumor stage (*p* = 0.008), N stage (*p* = 0.002), and survival outcome (*p* < 0.001). In addition, Student’s *t*-test analysis indicated that some of the signature-related genes were differentially expressed across various clinicopathological parameters (Figure S2, supporting information). We performed a univariate Cox regression analysis and a multivariate Cox regression analysis to verify the independent predictive value of the autophagy-related signature for OS. The univariate Cox analysis discovered that the autophagy-related signature, tumor stage, and T and N stages were all correlated with the survival of HNSCC patients (Fig. 3e). Then, those factors were included in a multivariate Cox analysis, and the results proved the predictive function of the autophagy-related signature in prognosis (Fig. 3f). Thus, our results confirmed that the autophagy-related signature could be used as an independent prognostic factor in clinical practice.

### Validation of the autophagy-related signature via an independent cohort

**Fig. 5:**
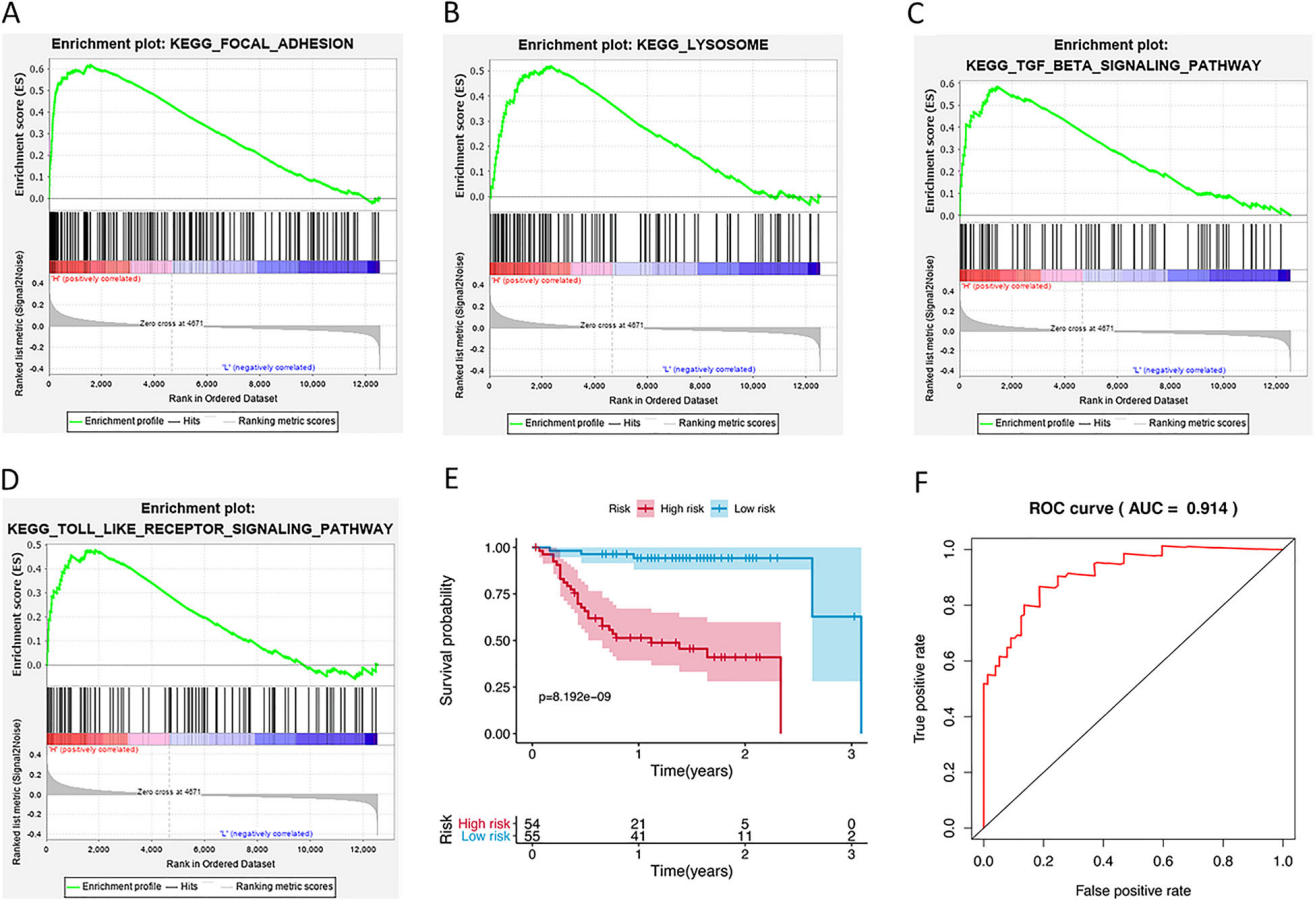
Validation of the autophagy-related signature in GEO dataset. **a** GSEA validated enhanced activity of Focal adhesion. **b** GSEA validated enhanced activity of Lysosome. **c** GSEA validated enhanced activity of TGF-β signaling pathway. **d** GSEA validated enhanced activity of Toll-like receptor signaling pathway. H indicates high risk; L indicates low risk. **e** Kaplan–Meier curves of OS in the high-risk and the low-risk groups stratified by the autophagy-related signature in the GSE27020. **f** The ROC analysis in the GSE27020.

We calculated the risk score for each patient in the GEO dataset GSE27020 as an independent external validation using the same formula. The patients were divided into high-risk and low-risk groups based on the median risk score. Then GSEA analysis was performed to identify autophagy associated pathways in the GSE27020. The significantly enriched autophagy-related pathways included regulatory pathways of focal adhesion, TGF-β signaling pathway, lysosome, and Toll-like receptor signaling pathway (Fig. 5a–d). The Kaplan–Meier analysis again confirmed the prognostic ability of our signature (Fig. 5e). As expected, the high-risk patients had a shorter OS than the low-risk patients (2-year survival rate = 41.0% vs. 94.2%, *p* < 0.001). The ROCs, with the AUC value of 0.914, also proved the excellent survival prediction ability of the signature (Fig. 5f). Owing to the lack of clinical data such as gender and tumor N stage, ROC analysis of other clinical factors could not be performed. These validation experiments confirmed the outstanding ability of the risk signature we constructed in predicting the prognosis of HNSCC patients.

## Discussion

Autophagy is involved in regulating cellular homeostasis and turnover of organelles and long-lived proteins in normal cells. It is an effective mechanism for the removal of whole organelles of cells such as leaky or surplus mitochondria, some potentially toxic protein aggregates too large for proteasomal removal, and intracellular microbes^11^. However, autophagy is a “double-edged sword” as it can have opposing effects on the survival and death of cells in accordance with the cellular environment in situ. It has been reported to play a key role in tumorigenesis, progression aggressiveness, and therapeutic resistance of multiple cancers^12,13^. In HNSCC, autophagy is induced by various risk factors. Major risk factors such as tobacco and alcohol have been found to induce oxidative stress, leading to chronic inflammation and induction of enhanced level of autophagy and finally resulting in oncogenic mutations^14^. HPV, an emerging risk factor for HNSCC, might also induce autophagy by enhancing expression of ARGs^15^.

However, little is known about the role of autophagy in the pathogenesis and progression of HNSCC. Large-scale databases, such as TCGA and GEO, provide us with effective measures to explore gene signatures, thus enable us to achieve a better understanding of the relationship between autophagy and tumors. In this study, based on the existing gene data of patients with HNSCC, we first identified 38 differentially expressed ARGs based on the TCGA database. The GO and KEGG analyses of the differentially expressed ARGs showed that most of them were involved in the regulation of autophagy, process utilizing autophagic mechanism, apoptosis and HPV infection. To analyze HNSCC prognosis-related genes from the perspective of autophagy, we screened and identified 10 prognostic ARGs in univariate and multivariate Cox regression analyses. The identified 10 prognosis-related ARGs could be used to establish a prognostic signature, which clustered patients with HNSCC into two groups. The results showed that the OS time in the high-risk group was significantly lower than the low-risk group. Considering HNSCC tends to metastasize to lymph nodes before distant metastasis, and lymph node metastasis is closely associated with poor prognosis in patients with HNSCC^16,17^. In addition, the TCGA cohort lacks half of HNSCC patients’ data of tumor M stage. Therefore, we excluded the clinical data of M stage for further analyses. Cox regression analysis confirmed the independent prognostic value of the autophagy-related signature. The risk score of autophagy-related signature is correlated with survival outcome, tumor size and lymph node metastasis. This suggested that our score was an effective index for the pathogenesis and progression of the disease, and played a vital role in the prognosis of the patients with HNSCC.

All genes in the signature were strongly correlated with cancer, as previous experiments demonstrated. CAPN10 had the smallest HR (0.4859) among the negative regulatory genes with the HR <1. Esteban et al. reported that some CAPN10 alleles may be exerting a protective effect on HNSCC risk in the Spanish population^18^. There are six major driver genes for HNSCC: LAMP1, ATG5, SAR1A, RAB24, ATIC, and ST13. In a previous study, oversialylated LAMP1 has been reported to enhance lysosomal exocytosis, making cancer cells migratory and invasive^19^. Moreover, GSEA analysis analyzed the differences between high- and low-risk groups stratified by the autophagy-related signature. We identified several pathways, such as “Focal adhesion” “Lysosome”, “TGF-β signaling pathway” and “Toll-like receptor signaling pathway”, which were significantly enriched in the high-risk group. It was also suggested that the autophagy-related signature might be concerned with HNSCC-related biological pathways and their functional dysregulations could lead to HNSCC relapse in GSEA enrichment results. Focal adhesion kinase (FAK) plays a key role in the regulation of cell migration, invasion, anchorage-independent growth, and anoikis resistance during cancer cell metastasis^20^. It has been reported that FAK overexpression is correlated with the invasive potential and lymph node metastasis in HNSCC^21^. TGF-β has also been found to be highly expressed in HNSCC which could increase the survival rate of fibroblasts, enhance cell proliferation and induce lymph node metastasis^22,23^. The lysosome is essential to autophagy, it has been mostly relegated to a role secondary to the autophagosome in studies on macroautophagy^24^. Kaplan–Meier curves of OS and ROC analyses in the GSE27020 validated that autophagy-related signature could be an independent prognostic indicator.

In conclusion, we established a model of autophagy-related gene signature that could be used to analyze the prognosis of patients with HNSCC, and validated by an independent cohort from the GEO database. Our study provided a new understanding of autophagy status in HNSCC, and the application of the signature in clinical treatments should also be further observed to verify the validity of our findings.

## Materials and methods

### Human autophagy-related gene (ARGs) set

We searched the Human Autophagy Database (HADb, http://autophagy.lu/clustering/index.html)^25^ to identify 232 genes involved in autophagy. The genes of autophagy process in HADb were shown in Table S1.

### Samples and data collected for this study

The HNSCC RNA sequencing (RNA-seq) expression data and corresponding clinical follow-up information were obtained from the public database TCGA. In total, there were clinical information of 528 patients and RNA-seq expression data of 546 samples. After excluding the normal patients and ones with less than 30 days’ follow-up, 519 patients’ expression data accompanied by their clinical information were extracted for our research. An independent microarray HNSCC cohort was extracted from the GEO database (accession number: GSE27020). There were data from 109 samples for clinical information and RNA-seq expression in the GSE27020. Log2 transformation was performed to analyze the expression data. ARGs associated with patients’ survival were identified using univariate Cox regression for subsequent model construction.

### Bioinformatics analysis

A Consensus Clustering Analysis and a Principle Components Analysis were performed by the R programming language (version 3.6) to verify the regulatory role of autophagy in HNSCC. The R package limma was used to screen the differentially expressed autophagy-related genes (Table S2). Then, we carried out a series of gene functional enrichment analyses to determine the major biological attributes, including the GO, KEGG, and GSEA analyses. The GOplot package was employed to visualize the enrichment terms. A univariate Cox proportional hazard regression analysis was applied to evaluate the association between overall survival (OS) and gene expression values. Next, a multivariate Cox proportional hazards regression analysis was performed using the candidate prognostic genes identified by the univariate regression analysis. The independent prognostic factors were determined by the multivariate Cox proportional hazards regression analyses, the regression coefficient and hazard ratios (HRs) were calculated by the Cox regression model.

### Construction of ARGs related prognostic model

Prognosis-related genes were constructed using multivariate cox regression. After incorporating the expression values for each particular gene, a risk score formula for each patient was constructed and weighted by its estimated regression coefficients in a multivariate cox regression analysis (Table S3). According to the risk scoring formula, the median risk score was used as the cut-off point, and the patients were divided into low-risk group and high-risk group. Survival differences between the two groups were assessed by Kaplan–Meier method and compared using log-rank statistical methods. Multivariate cox regression analysis and stratified analysis were used to examine the role of risk scores in predicting patient outcomes. ROC curves were used to study the accuracy of model predictions.

### Statistical analysis

Survival curves were generated by the Kaplan–Meier method and compared by log-rank test. Multivariate analysis was performed using the cox proportional hazard model. All statistical analyses were performed using the R language. All statistical tests were bilateral, with *p* < 0.05 being statistically significant.

## Supplementary information

Supporting Information.

Supporting Information.

Supplementary Information.

Supplementary Information.

Supplementary Information.

Supplementary Figures.
