# Supplementary figures and images for: Development and validation of prognostic index based on autophagy-related genes in patient with head and neck squamous cell carcinoma
Source: Cell Death Discov. 2020 Jul 14;6:59. doi: 10.1038/s41420-020-00294-y (PMC7360573; doi:10.1038/s41420-020-00294-y)

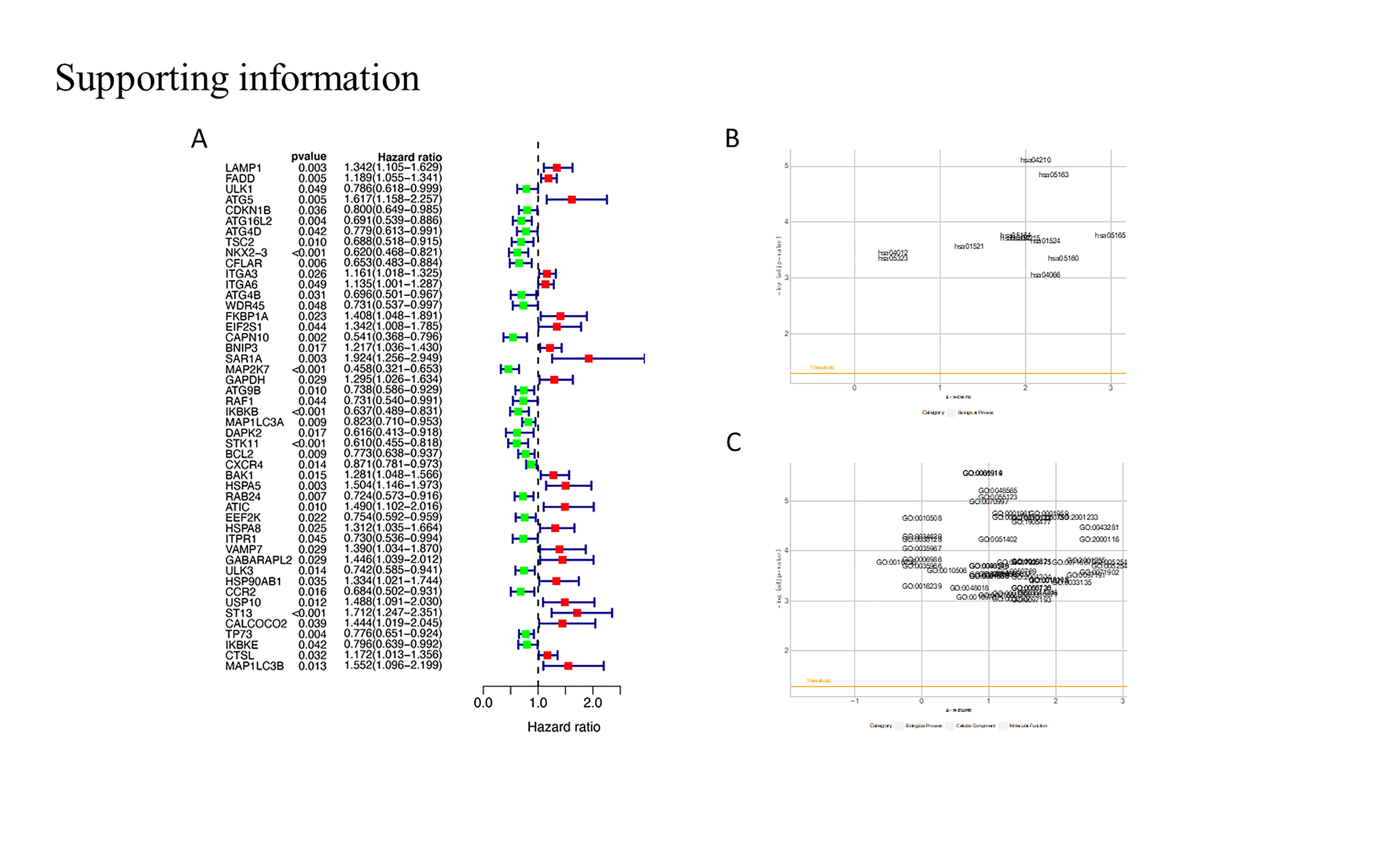

Supplement: Supplementary file 1 — Supporting Information. [file 41420_2020_294_MOESM1_ESM.png]

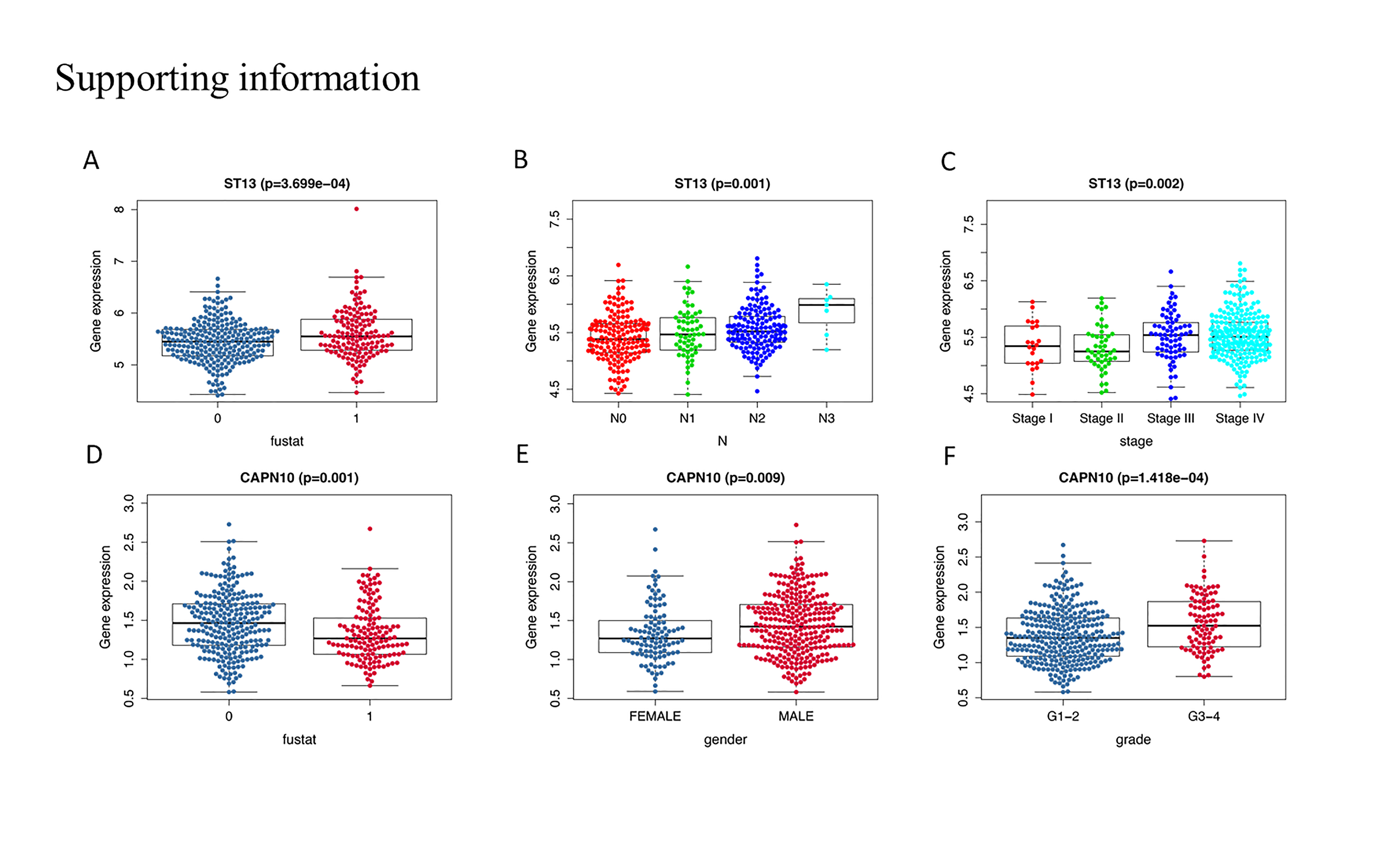

Supplement: Supplementary file 2 — Supporting Information. [file 41420_2020_294_MOESM2_ESM.png]
