# Supplementary material for: Development and validation of prognostic index based on autophagy-related genes in patient with head and neck squamous cell carcinoma
Source: Cell Death Discov. 2020 Jul 14;6:59. doi: 10.1038/s41420-020-00294-y (PMC7360573; doi:10.1038/s41420-020-00294-y)
Supplement: Supplementary file 6 — Supplementary Figures. [file 41420_2020_294_MOESM6_ESM.docx]

Figure S1 Expression profile and prognostic value of ARGs. (A) Risk ratio forest plot showed the prognostic value of the differentially expressed ARGS. (B) KEGG shows the signaling pathways involved in 38 differentially expressed ARGs. (C) GO analysis revealed the biological processes, molecular functions and cellular components involved in 38 differentially expressed ARGs.

Figure S2 The signature-related genes in the cohorts. (A, B, C) ST13 in the cohorts stratified by survival outcome, N stages and tumor stages (D, E, F) CAPN10 in the cohorts stratified by survival outcome, gender and grade.
